# Sources of Added Sugars in Young Children, Adolescents, and Adults with Low and High Intakes of Added Sugars

**DOI:** 10.3390/nu10010102

**Published:** 2018-01-17

**Authors:** Regan L. Bailey, Victor L. Fulgoni, Alexandra E. Cowan, P. Courtney Gaine

**Affiliations:** 1 Department of Nutrition Science, Purdue University, Stone Hall, Room 143A, 700 West State Street, West Lafayette, IN 47906, USA; 2 Nutrition Impact LLC, 9725 D Drive North, Battle Creek, MI 49014, USA; vic3rd@aol.com; 3 Department of Nutrition Science, Purdue University, Room 143, 700 West State Street, West Lafayette, IN 47906, USA; cowan9@purdue.edu; 4 The Sugar Association, Inc., 1300 L Street, NW, Suite 1001, Washington, DC 20005, USA; gaine@sugar.org

**Keywords:** NHANES, added sugars, obesity, sweetened beverages, Dietary Guidelines

## Abstract

High intake of added sugars is associated with excess energy intake and poorer diet quality. The objective of this cross-sectional study (*n* = 16,806) was to estimate usual intakes and the primary food sources of added sugars across the range of intakes (i.e., deciles) among U.S. children (2–8 years), adolescents and teens (9–18 years), and adults (≥19 years) using the National Health and Nutrition Examination (NHANES) data from 2009–2012. The percent energy contributed by added sugars was 14.3 ± 0.2% (2–8 years), 16.2 ± 0.2% (9–18 years), and 13.1 ± 0.2% (≥19 years), suggesting the highest intakes are among adolescents and teens. However, the primary foods/beverages that contribute to added sugars were remarkably consistent across the range of intakes, with the exception of the lowest decile, and include sweetened beverages and sweet bakery products. Interestingly across all age groups, even those in the lowest decile of added sugars exceed the 10% guidelines. Additional foods contributing to high intakes were candy and other desserts (e.g., ice cream) in children and adolescents, and coffee and teas in adults. Tailoring public health messaging to reduce intakes of these identified food groups may be of utility in designing effective strategies to reduce added sugar intake in the U.S.

## 1. Introduction

The dramatic increase in the prevalence of overweight and obesity has been a chief public health concern among U.S. adults, adolescents, and children [1,2]. Suboptimal weight status has tremendous negative health consequences and contributes to the most common chronic diseases of public health concern like cardiovascular disease (CVD), some cancer types, and type 2 diabetes [3]. Identifying contributing factors to overweight and obesity has been an active area of scientific investigation. High intake of dietary sugars has been identified not only with higher energy intakes, but also with diets of lower nutritional quality [4,5]. 

Many different types of dietary sugars exist but can loosely be broken down into those intrinsic to foods (i.e., naturally occurring) and those that are added for taste, food preservation, or other functional properties [6,7]. Intrinsic sugars are generally found in foods with favorable nutrient profiles such as fruits, vegetables, nuts, and dairy, whereas added sugars are often associated with foods and beverages lower in nutrient density. Examples of added sugars are sucrose, brown sugar, high fructose corn syrup, corn syrup, agave, dextrose, fructose, raw sugar, honey, invert sugar, maple syrup, concentrated fruit juice, and molasses [7,8]. Recommendations for intake of added sugars vary. The Dietary Guidelines for Americans recommend <10% of total energy to come from added sugars [9], whereas the Dietary Reference Intake (DRI) suggests <25%, although there is not a DRI for intake of added sugars [10]. The World Health Organization recommendations are for <10% of total energy from “free sugars”, which include both added sugars and all sugars present in 100% fruit juice [11]. Despite similar recommendations, the difference between “free sugars” and “added sugars” may lead to significantly different implications in specific age groups who regularly consume 100% fruit juice (i.e., young children). 

The percent of daily energy from added sugars has declined among U.S. adults from 18% in the NHANES 1999–2000 to 15% in NHANES 2007–2008 and more recently 13% in NHANES 2007–2010 but still remain above the national and global targets [12,13]. Similar decreases in intakes of added sugars have been documented among children, adolescents and teens [14]; however, mean energy intakes from added sugars tend to be higher (~16% of total energy) in the adolescent and teen age group [15]. 

It has been well documented that sweetened beverages are a leading source of added sugars across all age groups in the U.S. [15,16,17,18,19,20]. However, little is known about other top contributors, particularly as they may differ across range of intakes (e.g., from low to high intakes). Understanding the patterns across the continuum of added sugars intake would permit identification of potential strategies for intervention and help tailor public health messaging in order to meet the <10% of energy recommendations. The objective of this research was to estimate usual intake of added sugars, as well as food sources of added sugars, in both children and adults stratified by level of added sugars intake using nationally-representative survey data from the 2009–2012 NHANES.

## 2. Materials and Methods 

### 2.1. Data Source and Participants 

NHANES is a nationally-representative cross-sectional survey to assess the nutrition and health status of the U.S. resident population. What We Eat in America (WWEIA), the dietary interview component of NHANES 2009–2012, data were used for all analyses of children 2–8 years, adolescents and teens 9–18 years, and adults ≥19 years. The data consisted of two 24-h dietary recalls collected using an Automated Multiple-Pass Method [21], with the first recall conducted in-person in the Mobile Examination Center and the second recall conducted over the telephone. Proxies provided the 24-h dietary recalls for children 2–5 years of age; children aged 6–11 years were assisted by an adult, and older adolescents and teens provided their own recalls. Details of the survey design, protocols and data collection procedures are reported elsewhere in detail [22]. Pregnant and lactating women were excluded as well as those with incomplete and unreliable diet records as determined by USDA food research staff. The final analytic sample was comprised of 16,806 Americans ages 2–8 years (*n* = 2871), 9–18 years (*n* = 3238), and ≥19 years (*n* = 10,697). 

### 2.2. Added Sugars Intake 

Added sugars intake was determined using the USDA Food Patterns Equivalent Database (FPED) for each NHANES release (Table 1) [23]. FPED defines added sugars as “sugars that are added to foods as an ingredient during preparation, processing, or at the table” and does not include intrinsic sugars “such as lactose present in milk and fructose present in whole or cut fruit, and 100% fruit juice” [23]. However, for FPED 2011–2012, but not for FPED 2009–2010, fruit juice concentrates are also assigned to added sugars. Flavored milks would have both an intrinsic and added sugars component; however, only the added sugars component is part of this analysis. The 10% of total energy intake recommendation from the 2015–2020 Dietary Guidelines for Americans in used for reference in this analysis, but the NHANES data presented here precede this guidance. 

### 2.3. Statistical Analysis 

Data were analyzed using SAS 9.2 (SAS Institute, Cary, NC, USA) and SUDAAN release 11.0 (Research Triangle Institute, Research Triangle Park, NC, USA). Appropriate weighting factors and survey design features were used to adjust for oversampling of selected groups, and non-response of some individuals in order to make results nationally-representative. The National Cancer Institute (NCI) method was used to estimate usual intake of added sugars using both days of 24-h recall with a single component model since added sugars were consumed on most days by most subjects [24]. For both the percent energy from added sugars and grams per day, usual added sugars intake and deciles of intake were determined. Within each decile, sources of added sugars were determined and ranked using WWEIA food groups and categories. Mean estimates and the associated standard errors (SE) of added sugars were determined using PROC DESCRIPT of SUDAAN (which uses Taylor-series linearization for SE determination). Because of the descriptive nature of this analysis, no statistical comparisons were made.

## 3. Results

The mean percent energy contributed by added sugars was 14.3 ± 0.2% for those 2–8 years, 16.2 ± 0.2% for those 9–18 years, and 13.1 ± 0.2% for those ≥19 years. Across all groups, the first decile intakes ranged between 11–13% of total energy contributed from added sugars whereas in the highest decile, this range hovered around 18–20% (Table 2). The range of added sugars intake from decile 1 to decile 10 was narrower among children 2–8 years and adolescents and teens 9–18 years (i.e., ~2 times, from less than 10 tsp/day (40 g) to more than 20 tsp/day (80 g) in 2–8 y and <15 tsp/day (60 g) to 27 tsp/day (108 g) in 9–18 years) than in adults where the range was wider (from less than 8 tsp/day (32 g) to equal to or greater than 31 tsp/day (124 g) in adults; i.e., 3.7 times). 

### 3.1. Children 2–8 Years 

Among children in the lowest decile, added sugars intake was primarily coming from intakes of ready-to-eat cereals (14.8%), sweet bakery products (14.7%) and sweetened beverages (12.4%) (Table 3). In the mid-range of added sugars intake (decile 5), sweetened beverages (22.8%) ranked first, followed by sweet bakery products (16.1%) and “other” desserts (8.8%). At the highest level of intake (decile 10), sweetened beverages (36.8%) ranked first, sweet bakery products (14.3%) ranked second, and candy (9.1%) ranked third. While many of the same food groups contributed to added sugars across all levels of intake, flavored milk and snack/meal bars were only among the top ten in children in the lowest decile of added sugars intake. The amount of added sugars contributed by sweetened beverages increased dramatically across deciles of intake (Table S1). Within this category, fruit drinks were the highest source of added sugars consumed, followed by soft drinks. Coffee and tea products appear as a top ten source of added sugars in deciles 6 and 8–10, although the overall contribution was small (i.e., 1.7–3.3%). 

The contribution of sweet bakery products to added sugars was relatively stable across the deciles, ranging between 13–17%. Cookies and brownies were the most frequently consumed sweet bakery products. Ready-to-eat cereals showed a consistent drop in intake across deciles, ranking first (14.8%) in decile 1, ranking fifth (6.5%) in decile 5, and ranking seventh (4.3%) in decile 10. Candy intake increased across deciles, ranking seven (5.4%) in decile 1, ranking six in decile 5 (6.2%) and ranking third (9.1%) in decile 10.

### 3.2. Adolescents and Teens 9–18 Years

Among adolescents and teens, the three highest ranked foods in decile 1 were ready-to-eat cereals (12.5%), sweetened beverages (12.3%) and breads, rolls, tortillas (10.4%) in contrast to decile 10 where sweetened beverages (53.3%), sweet bakery products (11.4%) and candy (5.6%) were the highest ranked foods (Table 4). When compared to children 2–8 years, far fewer food groups substantially contributed (>5%) to intakes of added sugars, and this was more pronounced with increasing deciles suggesting that the high intakes in this age group are attributed to a few specific groups of foods. Sweetened beverages was the first ranked food category from decile 2 onwards, where it was contributing more than a quarter of added sugars with increases to more than half of added sugars in decile 10 (Table S2). Soft drinks were the primary contributor to the sweetened beverage category in all deciles, followed by fruit drinks. Soft drinks doubled the amount of added sugars contribution of fruit drinks in decile 5, steadily increasing to triple the added sugars contribution of fruit drinks in decile 10 (data not shown). The coffee and tea category contributed 4–6% of added sugars across deciles, ranking below the top ten foods in decile 2 but rising to rank 3 in both deciles 8 and 9. Sweetened tea products was a frequently consumed beverage in the coffee and tea category.

Sweet bakery products also were consistently an important source of added sugars. While this food group ranked fourth in decile 1, it was the second highest contributor to added sugars across all other deciles, contributing between 9–14% of all added sugars consumed. Within this group, the most commonly reported foods were cookies, brownies, cakes, and pies. The “other” desserts category contributed between 5–10% of added sugars intake across deciles, and includes foods such as ice cream, frozen dairy desserts, puddings, gelatins, ices and sorbets. Candy intake fluctuated between 5–8% of added sugars intake across all deciles and, as a proportion of total sugars intake, candy was highest in decile 2 and lowest in decile 6. Although candy ranked third in decile 10, it contributed only 5.6% of total added sugars in that group. Flavored milk ranked sixth, fifth and fourth, respectively, in deciles 1–3, fell to ninth in deciles 4 and 5 and stayed at eighth for deciles 6–10. However, the overall contribution of flavored milk to added sugars was not substantial (i.e., 1.5–6% across deciles). The rankings for ready-to-eat cereals ranged between first (12.5%, decile 1) and seventh (4%, decile 9) across deciles. 

### 3.3. Adults ≥ 19 Years

In decile 1, breads, rolls, tortillas (20.0%), sweet bakery products (10.4%), and fats and oils (9.7%, mainly in the form of salad dressing and condiments) were the highest contributors of added sugars in adults ≥19 years (Table 5). Sweetened beverages (23.1%), sweet bakery products (13.9%), and sugars (9.5%) were the three highest ranked food categories in decile 5. At the highest level of added sugars intake (decile 10), sweetened beverages (51.4%) was the highest contributor, followed by coffee and tea (10.6%) and sweet bakery products (9.5%). 

Sweetened beverages was ranked first from decile 5 to decile 10, contributing between almost a quarter to just over half of the daily added sugars intake (Table S3). In adults, the absolute contribution of soft drinks was much higher than in children, followed by fruit drinks across all ten deciles with the addition of sports and energy drinks in the higher deciles (i.e., decile 7 and up, data not shown). Sweet bakery products were the next most frequently consumed source of added sugars, ranking first or second for deciles 1 to 9 (10.4–15%) and ranking third for decile 10 (9.5%). Cakes, pies, cookies, and brownies were significant contributors in the sweet bakery category. 

Sweetened tea products in the coffee and tea category had relatively steady increases from decile 3, where they ranked ninth (3.5%) to decile 10 where they ranked second (10.6%). Ready-to-eat cereals decreased in rank from fourth in decile 1 to eighth in decile 5 and then ranked seventh from deciles 6 to 10. It contributed 5.3–7.9% of added sugars in deciles 1 to 6 and contributed 2.2–3.7% in deciles 7 to 10. The intakes of “other” desserts, particularly ice cream and frozen dairy desserts, increased from seventh in deciles 2 and 3 to third in decile 6, and then down to fifth of sixth in deciles 8–10; the overall contribution of ”other” desserts ranged between 5.7% to 8.2%. Sugars as a food group (i.e., syrups, honey, jams, etc.) ranked third or fourth between deciles 2 and 10, contributing 8–9.5% of added sugars intake. Candy contributed between 5.5–7.8% of added sugars intake across all deciles, with a high rank of fourth in decile 4 and a lower rank of eighth in decile 2. Candy had a rank of fifth or sixth in the remaining deciles.

## 4. Discussion

Our analysis of nationally-representative survey data affirms that intakes of added sugars continue to be well above authoritative recommendations; even the lowest deciles of intakes in children, adolescents and teens, and adults, on average, exceed the recommendation for less than 10% of calories from added sugars. These data also confirm that the primary source of added sugars for most of the U.S. population (≥2 years) is sweetened beverages. However, the type of beverages contributing to the sweetened beverage category changes with increasing intake of added sugars and also with age group. Among children, juice-based and fruit beverages are the key contributors, whereas in adolescents, teens and adults, soft drinks (i.e., sodas, pops, and colas) play this role. Surprisingly, sports and energy drinks were increasingly prevalent in the higher deciles of added sugars intake in both adolescents and teens, and adults, but to a greater degree in those 9–18 years.

Our results add to the growing body of literature that identifies intake of sweetened beverages, referred to in the literature as sugar-sweetened beverages (SSB), as being highly associated with added sugars intake, both in absolute and relative terms [14,25]. While Kit et al. have demonstrated with trend analysis that SSB consumption has slowly declined across age, sex, and race-ethnicity groups [26], SSB still contribute more than one-third (39%) of all added sugars consumed in the U.S. and compromise ~8% of total energy from added sugars in adolescents and adults, a number that hovers near the 10% recommendation [26]. Given that almost all SSB are high in calories and not dense in nutrients, their consumption has been associated with excess body weight [27,28], type 2 diabetes [29,30,31] and CVD [32,33]. However, Sievenpiper and de Souza [34] and others [7] have questioned if there is an independent role of sugars and SSBs or if sugars and SSBs simply add excessive calories to the diet contributing to these health conditions. Additionally, in this and other reports relative to SSBs, 100% fruit juice was not characterized as a sweetened beverage, as it would be if the World Health Organization definition of free sugars was used [11]. Nutrition scientists and pediatric health professionals acknowledge that consumption of 100% fruit juice contributes positively to nutrient intakes, but should be provided to children in appropriate portion sizes to reduce the risk of excess calories [7,35,36]. A recent National Academies report outlines the need for research surrounding 100% fruit juices [36]. Furthermore, flavored teas and coffees contributed (≥5% of total) in the higher deciles of intake of added sugars in both adolescents and teens, and adults. Thus, the role of beverages toward added sugars is probably larger than has been reported here and elsewhere because they are treated as two distinct food categories.

The second main source of added sugars across all age groups was sweet bakery products (i.e., cookies, brownies, cakes and pies, doughnuts, sweet rolls, and pastries). This finding is consistent with previous NHANES data that indicated that snacks and sweets contributed 31% of total intake of added sugars [9]. In addition to being a primary source of added sugars, sweet bakery products are also contributors of saturated fat intake in the American diet [37,38]. Thus, public health messaging surrounding reduced intake of added sugars by reductions in sweet bakery products has the potential to reduce saturated fat intakes, also known to be above the Dietary Guidelines recommendations.

Sugars and candy were among the top ten sources of added sugars in all deciles across all age groups. Among children, and adolescents and teens, trends of candy intake generally increased across deciles of added sugars. This trend was less apparent in adults where candy was ranked fifth or sixth in most deciles. Sugars as a category represent different dimensions of the diet than foods consumed alone (i.e., candy, yogurt) and are therefore difficult to make concrete recommendations about because they have the potential to be consumed in unhealthy and healthy (e.g., added to oatmeal) ways. In contrast to empty calorie foods, food categories that contribute to added sugars, such as ready-to-eat cereals, flavored milk, and yogurt, are also important sources of nutrients such as dietary fiber and vitamins and minerals. All three of these food categories were top ten sources of added sugars in children 2–8 years, with ready-to-eat cereals ranking first in decile 1 and dropping to rank seven in decile 10. Ready-to-eat cereals showed a similar pattern in adolescents and teens 9–18 years. Flavored milk was a top ten source of added sugar in adolescents and teens, whereas yogurt was not. Among adults, ready-to-eat cereals were a top ten source, but were less prominent than in the diets of children. Flavored milk and yogurt were not important sources of added sugars in adults. 

Our results are generally concordant with the findings of earlier analyses of intake of added sugars. The major sources of calories from added sugars in U.S. youth 2–18 years in NHANES 2003–2004 were from soda (116 kcal/day), fruit drinks (55 kcal), grain desserts (40 kcal), dairy desserts (29 kcal), and candy (25 kcal) [25]. Analysis of the entire U.S. population (≥2 years) has indicated that soft drinks were the largest contributor of added sugars in the last decade, followed by cakes and cookies, fruit ades and sport drinks, sugars and syrups, and candies and gum [13]. However, these studies differed from ours in both the age of participants as well as some food categories. 

The strengths of our study include a large sample size, the use of food groups and food categories defined by WWEIA, a nationally representative database for determining added sugars, and the use of the NCI method to estimate usual or habitual consumption patterns. Limitations include the use of self-reported dietary recalls that may underestimate actual intake [39,40]. Another consideration is that foods and beverages consumed by children in daycare and schools may or may not be accurately reported by the proxies. Development of a biomarker to assess intakes or sugars would be beneficial to better estimate intakes; fortunately, research in this area is underway [41]. 

## 5. Conclusions

Public health efforts to reduce intake of added sugars should put the greatest emphasis on decreasing the amount of sweetened beverages consumed first and foremost, followed by sweet bakery products. Depending on age, specific messaging about intakes of candy and other desserts is warranted. Food manufacturers should also be encouraged to decrease the added sugars content of food products without contributing to excess calories [42]. However, all sectors have roles to play in improving diet quality and the most recent Dietary Guidelines highlight the need for this social ecological approach [9]. The Dietary Guidelines also recommend small changes be made to improve health. For example, based on our data, some small changes that could be recommended include the substitution of water or milk for SSB. Dietitians have long heralded “watering down” sugary drinks before providing them to children, if providing them at all. Clearly, another point of intervention can focus on baked goods; here, recommending smaller portions or substituting more healthy alternatives may be helpful. 

In conclusion, there were no major differences in the types of foods that low consumers of added sugars choose versus those who were high consumers within the three age groups we analyzed. The main differences were shifts in the amounts of those specific foods consumed across deciles. This data can be used to inform age-specific recommendations to improve diets of Americans. Awareness of these trends in foods that contribute to added sugars is useful in designing effective strategies to reduce intakes of added sugars in the U.S. population to comply with the Dietary Guidelines recommendations. 

## Figures and Tables

**Table 1:** Breakdown of food groups that contribute to intakes of added sugars determined by What We Eat in America (WWEIA).

Food Group	Food Types in Food Groups
Breads, Rolls, Tortillas	Yeast breads, rolls and buns, bagels, English muffins, tortillas
Candy	Candy containing chocolate, candy not containing chocolate
Coffee and Tea	Coffee and tea
Condiments and Sauces	Tomato-based condiments, soy-based condiments, mustard and other condiments, olives, pickles, pickled vegetables, pasta sauces including tomato-based, dips, gravies, other sauces
Cured Meats/Poultry	Cold cuts and cured meats, bacon, frankfurters, sausages
Fats and Oils	Butter, animal fats, margarine, cream cheese, sour cream, whipped cream, cream and cream substitutes, mayonnaise, salad dressings, vegetable oils
Flavored Milk	Flavored milk-whole, reduced fat, low fat, nonfat
Mixed Dishes-Pizza	Mixed dishes and pizza, all varieties
Mixed Dishes-Sandwiches	Burgers, frankfurter sandwiches, chicken/turkey sandwiches, egg breakfast sandwiches, other sandwiches
Other Desserts	Ice cream, frozen dairy desserts, pudding, gelatins, ices, sorbets
Quick Breads and Bread Products	Biscuits, muffins, quick breads, pancakes, waffles, French toast
Ready-to-Eat Cereals	Ready to eat cereals, higher sugar (>21.2 g/100 g), RTE cereals, lower sugar (≤21.2 g/100 g)
Snack/Meal Bars	Cereal bars, nutrition bars
Sugars	Sugars, honey, sugar substitutes, jams, syrups, toppings
Sweet Bakery Products	Cakes, pies, cookies, brownies, doughnuts, sweet rolls, pastries
Sweetened Beverages	Soft drinks, fruit drinks, sport and energy drinks, nutritional beverages, smoothies and grain drinks
Yogurt	Yogurt regular, Greek

**Table 2:** Usual added sugars intake by decile of intake by age groups, data from the National Health and Nutrition Survey, 2009–2012 ^1^.

Decile	2–8 Years		9–18 Years		≥19 Years	
		g/Day	% kcal/Day	g/Day	% kcal/Day	g/Day	% kcal/Day
1	<42.8	<11.2	<59.9	<12.8	<33.0	<11.9
2	42.8–49.1	11.2–12.3	59.9–65.7	12.8–13.9	33.0–42.4	11.9–13.2
3	49.2–54.0	12.4–13.2	65.8–71.5	14.0–14.9	42.5–50.1	13.3–14.0
4	54.1–58.7	13.3–13.8	71.6–76.4	15.0–15.6	50.2–57.9	14.1–14.9
5	58.8–63.1	13.9–14.6	76.5–80.9	15.7–16.4	58.0–66.2	15.0–15.6
6	63.2–67.7	14.7–15.2	81.0–86.0	16.5–17.2	66.3–75.1	15.7–16.4
7	67.8–72.9	15.3–15.9	86.1–92.6	17.3–17.9	75.2–85.9	16.5–17.2
8	73.0–77.5	16.0–16.8	92.7–100.1	18.0–18.8	86.0–99.8	17.3–18.1
9	77.6–86.1	16.9–18.1	100.2–110.8	18.9–20.3	99.9–123.7	18.2–19.5
10	≥86.2	≥18.2	≥110.9	≥20.4	≥123.8	≥19.6

^1^ Usual added sugars intake determined using the National Cancer Institute.

**Table 3:** Top ten food sources of added sugars for children 2–8 years by decile of intake, data from the National Health and Nutrition Survey, 2009–2012 (*n* = 2871).

Rank	Decile 1		Decile 5		Decile 10	
	Food Group	% Total Added Sugars from Food Sub-Group ^1^	Food Sub-Group	% Total Added Sugars from Food Sub-Group	Food Sub-Group	% Total Added Sugars from Food Sub-Group
1	Ready-to-Eat Cereals	14.8 ± 1.0	Sweetened Beverages	22.8 ± 2.2	Sweetened Beverages	36.8 ± 2.4
2	Sweet Bakery Products	14.7 ± 1.7	Sweet Bakery Products	16.1 ± 2.0	Sweet Bakery Products	14.3 ± 1.6
3	Sweetened Beverages	12.4 ± 2.1	Other Desserts	8.8 ± 1.7	Candy	9.1 ± 1.5
4	Other Desserts	7.7 ± 1.5	Flavored Milk	7.7 ± 1.7	Other Desserts	7.6 ± 1.1
5	Breads, Rolls, Tortillas	7.3 ± 1.2	Ready-to-Eat Cereals	6.5 ± 1.0	Flavored Milk	5.7 ± 1.0
6	Sugars	6.6 ± 1.0	Candy	6.3 ± 1.2	Sugars	4.8 ± 0.6
7	Candy	5.4 ± 1.2	Sugars	5.9 ± 1.6	Ready-to-Eat Cereals	4.3 ± 0.7
8	Yogurt	4.2 ± 1.5	Yogurt	5.5 ± 1.0	Coffee and Tea	3.3 ± 1.8
9	Quick Breads and Bread Products	3.4 ± 0.6	Breads, Rolls, Tortillas	3.0 ± 0.5	Yogurt	2.6 ± 1.0
10	Snack/Meal Bars	3.1 ± 0.9	Quick Breads and Bread Products	2.6 ± 0.6	Quick Breads and Bread Products	1.8 ± 0.5

^1^ Mean ± standard error.

**Table 4:** Top ten food sources of added sugars for adolescents and teens 9–18 years by decile of intake, data from the National Health and Nutrition Survey, 2009–2012 (*n* = 3238) ^1^.

Rank	Decile 1		Decile 5		Decile 10	
	Food Group	% Total Added Sugars from Food Sub-Group	Food Sub-Group	% Total Added Sugars from Food Sub-Group	Food Sub-Group	% Total Added Sugars from Food Sub-Group
1	Ready-to-Eat Cereals	12.5 ± 2.4	Sweetened Beverages	33.1 ± 2.7	Sweetened Beverages	53.3 ± 1.7
2	Sweetened Beverages	12.3 ± 1.4	Sweet Bakery Products	9.4 ± 1.6	Sweet Bakery Products	11.4 ± 1.1
3	Breads, Rolls, Tortillas	10.4 ± 1.7	Other Desserts	7.1 ± 1.7	Candy	5.6 ± 1.1
4	Sweet Bakery Products	8.0 ± 1.0	Sugars	6.7 ± 3.4	Other Desserts	5.5 ± 0.8
5	Mixed Dishes–Pizza	6.7 ± 1.7	Ready-to-Eat Cereals	6.1 ± 1.2	Coffee and Tea	4.9 ± 1.2
6	Flavored Milk	6.3 ± 1.6	Coffee and Tea	5.9 ± 2.1	Ready-to-Eat Cereals	4.7 ± 1.0
7	Other Desserts	5.6 ± 1.4	Candy	4.2 ± 1.0	Sugars	3.1 ± 0.5
8	Candy	5.2 ± 1.1	Breads, Rolls, Tortillas	4.0 ± 0.9	Flavored Milk	1.5 ± 0.3
9	Sugars	4.5 ± 1.1	Flavored Milk	3.9 ± 0.7	Breads, Rolls, Tortillas	1.4 ± 0.2
10	Coffee and Tea	4.1 ± 1.2	Quick Breads and Bread Products	3.5 ± 0.8	Quick Breads and Bread Products	1.2 ± 0.4

^1^ Mean ± standard error.

**Table 5:** Top ten food sources of added sugars for adults ≥19 years by decile of intake, data from the National Health and Nutrition Survey, 2009–2012 (*n* = 10,697) ^1^.

Rank	Decile 1		Decile 5		Decile 10	
	Food Group	% Total Added Sugars from Food Sub-Group	Food Sub-Group	% Total Added Sugars from Food Sub-Group	Food Sub-Group	% Total Added Sugars from Food Sub-Group
1	Breads, Rolls, Tortillas	20.0 ± 0.9	Sweetened Beverages	23.1 ± 1.4	Sweetened Beverages	51.4 ± 1.5
2	Sweet Bakery Products	10.4 ± 0.9	Sweet Bakery Products	13.9 ± 1.1	Coffee and Tea	10.6 ± 1.0
3	Fats and Oils	9.7 ± 0.9	Sugars	9.5 ± 0.6	Sweet Bakery Products	9.5 ± 0.6
4	Ready-to-Eat Cereals	7.2 ± 1.0	Other Desserts	6.8 ± 1.0	Sugars	6.4 ± 0.4
5	Sugars	5.8 ± 0.8	Candy	6.6 ± 0.9	Candy	4.8 ± 0.4
6	Candy	5.5 ± 1.1	Coffee and Tea	6.1 ± 0.7	Other Desserts	4.5 ± 0.6
7	Sweetened Beverages	4.4 ± 0.8	Breads, Rolls, Tortillas	5.8 ± 0.5	Ready-to-Eat Cereals	2.2 ± 0.2
8	Condiments and Sauces	3.8 ± 0.6	Ready-to-Eat Cereals	5.3 ± 0.7	Fats and Oils	1.2 ± 0.2
9	Mixed Dishes-Sandwiches	3.2 ± 0.7	Fats and Oils	3.4 ± 0.4	Breads, Rolls, Tortillas	1.1 ± 0.1
10	Cured Meats/Poultry	2.8 ± 0.5	Snack/Meal Bars	2.0 ± 0.4	Quick Breads and Bread Products	1.1 ± 0.2

^1^ Mean ± standard error.

## Supplementary Materials

The following are available online at www.mdpi.com/2072-6643/10/1/102/s1, Table S1: Top Ten Food Sources of Added Sugars for Children 2–8 years by Ten Deciles of Intake, Table S2: Top Ten Food Sources of Added Sugars for Children and Adolescents 9–18 years by Ten Deciles of Intake, and Table S3: Top Ten Food Sources of Added Sugars for Adults ≥19 years by Ten Deciles of Intake.
