# Supplementary material for: Sources of Added Sugars in Young Children, Adolescents, and Adults with Low and High Intakes of Added Sugars
Source: Nutrients. 2018 Jan 17;10(1):102. doi: 10.3390/nu10010102 (PMC5793330; doi:10.3390/nu10010102)
Supplement: Supplementary file 1 [file nutrients-10-00102-s001.pdf]

nutrients

MDPI

Appendix A: Supplemental Tables

Table S1. Top Ten Food Sources of Added Sugars for Children 2-8 y by Ten Deciles of Intake[1]

Rank	Decile 1		Decile 2		Decile 3		Decile 4		Decile 5		Decile 6		Decile 7		Decile 8		Decile 9		Decile 10	
	Food Sub Group	% Total Added Sugars	Food Sub Group	% Total Added Sugars	Food Sub Group	% Total Added Sugars	Food Sub Group	% Total Added Sugars	Food Sub Group	% Total Added Sugars	Food Sub Group	% Total Added Sugars	Food Sub Group	% Total Added Sugars	Food Sub Group	% Total Added Sugars	Food Sub Group	% Total Added Sugars	Food Sub Group	% Total Added Sugars
1	RTE Cereals	14.8 ± 1.00	Sweetened Beverages	16.6 ± 1.94	Sweet Bakery Products	15.2 ± 1.95	Sweetened Beverages	17.3 ± 1.83	Sweetened Beverages	22.8 ± 2.18	Sweetened Beverages	25.2 ± 2.94	Sweetened Beverages	31.1 ± 2.57	Sweetened Beverages	29.0 ± 2.49	Sweetened Beverages	33.4 ± 2.14	Sweetened Beverages	36.8 ± 2.44
2	Sweet Bakery Products	14.7 ± 1.69	Sweet Bakery Products	12.7 ± 1.38	Sweetened Beverages	14.8 ± 2.11	Sweet Bakery Products	14.1 ± 1.81	Sweet Bakery Products	16.1 ± ± 2.03	Flavored Milk	12.6 ± 1.80	Sweet Bakery Products	12.1 ± 1.16	Sweet Bakery Products	16.6 ± 1.64	Sweet Bakery Products	14.9 ± 1.69	Sweet Bakery Products	14.3 ± 1.58
3	Sweetened Beverages	12.4 ± 2.14	RTE Cereals	12.5 ± 1.47	RTE Cereals	11.1 ± 2.21	RTE Cereals	12.9 ± 1.43	Other Desserts	8.8 ± 1.69	Sweet Bakery Products	11.9 ± 1.62	Other Desserts	8.2 ± 1.12	Other Desserts	7.5 ± 1.71	Other Desserts	8.5 ± 1.39	Candy	9.1 ± 1.48
4	Other Dessert	7.7 ± 1.51	Other Desserts	8.1 ± 1.09	Flavored Milk	7.8 ± 1.61	Flavored Milk	8.8 ± 1.66	Flavored Milk	7.7 ± 1.71	RTE Cereals	10.4 ± 1.37	Flavored Milk	8.1 ± 1.01	Candy	7.5 ± 1.24	Sugars	7.4 ± 3.25	Other Desserts	7.6 ± 1.08
5	Breads, Rolls, Tortillas	7.3 ± 1.16	Yogurt	6.8 ± 1.27	Other Desserts	7.3 ± 1.35	Other Desserts	8.8 ± 1.39	RTE Cereals	6.5 ± 0.96	Other Desserts	6.3 ± 1.41	Candy	7.4 ± 1.18	Flavored Milk	7.3 ± 0.98	Candy	7.1 ± 1.31	Flavored Milk	5.7 ± 0.98
6	Sugars	6.6 ± 1.03	Sugars	6.0 ± 0.89	Sugars	6.8 ± 1.17	Sugars	5.3 ± 0.93	Candy	6.3 ± 1.19	Candy	6.2 ± 0.91	RTE Cereals	7.3 ± 0.96	RTE Cereals	6.6 ± 0.83	Flavored Milk	6.0 ± 0.94	Sugars	4.8 ± 0.63
7	Candy	5.4 ± 1.16	Candy	5.7 ± 0.94	Yogurt	5.2 ± 1.51	Yogurt	4.6 ± 1.37	Sugars	5.9 ± 1.55	Sugars	4.0 ± 0.66	Sugars	5.0 ± 1.06	Sugars	6.4 ± 1.04	RTE Cereals	5.8 ± 0.75	RTE Cereals	4.3 ± 0.68
8	Yogurt	4.2 ± 1.47	Bread, Rolls, Tortillas	4.8 ± 0.60	Bread, Rolls, Tortillas	4.7 ± 0.62	Candy	4.4 ± 0.80	Yogurt	5.5 ± 1.03	Coffee, Tea	3.1 ± 1.37	Breads, Rolls, Tortillas	2.8 ± 0.28	Yogurt	2.4 ± 0.93	Yogurt	2.4 ± 0.68	Coffee, Tea	3.3 ± 1.79
9	Quick Breads, Bread Products	3.4 ± 0.58	Flavored Milk	3.6 ± 0.70	Candy	4.3 ± 0.97	Quick Breads, Bread Products	3.7 ± 1.48	Breads, Rolls, Tortillas	3.0 ± 0.47	Quick Breads, Bread Products	2.8 ± 1.04	Yogurt	2.5 ± 0.76	Breads, Rolls, Tortillas	2.2 ± 0.22	Breads, Rolls, Tortillas	1.9 ± 0.24	Yogurt	2.6 ± 1.02
10	Snack/ Meal Bars	3.1 ± 0.92	Fruits	3.4 ± 0.85	Fruits	3.1 ± 0.82	Bread, Rolls, Tortillas	3.1 ± 0.30	Quick Breads, Bread Products	2.6 ± 0.61	Breads, Rolls, Tortillas	2.4 ± 0.24	Quick Breads, Bread Products	2.0 ± 0.41	Coffee, Tea	2.0 ± 0.69	Coffee, Tea	1.7 ± 0.49	Quick Breads, Bread Products	1.8 ± 0.51

[1]Mean ± standard error

Table S2. Top Ten Food Sources of Added Sugars for Children and Adolescents 9-18 y by Ten Deciles of Intake[1]

Rank	Decile 1		Decile 2		Decile 3		Decile 4		Decile 5		Decile 6		Decile 7		Decile 8		Decile 9		Decile 10	
	Food Sub Group	% Total Added	Food Sub Group	% Total Added	Food Sub Group	% Total Added	Food Sub Group	% Total Added	Food Sub Group	% Total Added	Food Sub Group	% Total Added	Food Sub Group	% Total Added	Food Sub Group	% Total Added	Food Sub Group	% Total Added	Food Sub Group	% Total Added
1	Ready-to-Eat Cereals	12.5 ± 2.44	Sweetened Beverages	27.4 ± 3.89	Sweetened Beverages	32.8 ± 2.77	Sweetened Beverages	30.1 ± 2.37	Sweetened Beverages	33.1 ± 2.74	Sweetened Beverages	40.1 ± 2.77	Sweetened Beverages	38.3 ± 2.66	Sweetened Beverages	44.2 ± 2.36	Sweetened Beverages	41.3 ± 2.28	Sweetened Beverages	53.3 ± 1.74
2	Sweetened Beverages	12.3 ± 1.41	Sweet Bakery Products	12.3 ± 1.81	Sweet Bakery Products	11.8 ± 1.53	Sweet Bakery Products	13.7 ± 2.12	Sweet Bakery Products	9.4 ± 1.55	Sweet Bakery Products	10.4 ± 1.07	Sweet Bakery Products	13.9 ± 1.67	Sweet Bakery Products	13.7 ± 1.93	Sweet Bakery Products	14.1 ± 1.37	Sweet Bakery Products	11.4 ± 1.08
3	Breads, Rolls, Tortillas	10.4 ± 1.66	Ready-to-Eat Cereals	10.5 ± 1.73	Ready-to-Eat Cereals	7.8 ± 1.04	Other Desserts	10.3 ± 1.92	Other Desserts	7.1 ± 1.69	Sugars	7.3 ± 2.51	Other Desserts	7.7 ± 1.32	Coffee, Tea	6.2 ± 1.21	Coffee, Tea	6.6 ± 1.22	Candy	5.6 ± 1.06
4	Sweet Bakery Products	8.0 ± 1.03	Candy	7.9 ± 2.04	Flavored Milk	6.2 ± 1.63	Ready-to-Eat Cereals	9.0 ± 1.26	Sugars	6.7 ± 3.43	Other Desserts	5.6 ± 1.31	Ready-to-Eat Cereals	6.4 ± 0.85	Other Desserts	6.0 ± 1.19	Candy	6.2 ± 1.44	Other Desserts	5.5 ± 0.80
5	Mixed Dishes – Pizza	6.7 ± 1.73	Flavored Milk	4.7 ± 1.41	Candy	6.0 ± 1.40	Coffee, Tea	5.5 ± 1.33	Ready-to-Eat Cereals	6.1 ± 1.19	Ready-to-Eat Cereals	5.3 ± 0.87	Candy	6.0 ± 1.26	Candy	5.5 ± 0.78	Other Desserts	6.1 ± 1.33	Coffee and Tea	4.9 ± 1.24
6	Flavored Milk	6.3 ± 1.61	Breads, Rolls, Tortillas	4.6 ± 0.75	Other Desserts	5.3 ± 1.09	Candy	5.3 ± 1.12	Coffee and Tea	5.9 ± 2.10	Candy	4.8 ± 0.83	Coffee, Tea	6.0 ± 1.63	Ready-to-Eat Cereals	4.5 ± 0.57	Sugars	5.2 ± 0.92	Ready-to-Eat Cereals	4.7 ± 1.00
7	Other Desserts	5.6 ± 1.40	Sugars	4.5 ± 1.16	Breads, Rolls, Tortillas	4.8 ± 0.71	Breads, Rolls, Tortillas	3.2 ± 0.27	Candy	4.2 ± 0.95	Coffee, Tea	4.6 ± 1.07	Sugars	4.7 ± 0.73	Sugars	3.8 ± 0.68	Ready-to-Eat Cereals	4.0 ± 0.66	Sugars	3.1 ± 0.53
8	Candy	5.2 ± 1.12	Other Desserts	4.4 ± 0.78	Sugars	3.2 ± 0.66	Dairy Drinks & Substitutes	2.8 ± 1.31	Breads, Rolls, Tortillas	4.0 ± 0.88	Flavored Milk	4.4 ± 1.07	Flavored Milk	4.5 ± 1.05	Flavored Milk	3.3 ± 0.79	Flavored Milk	1.9 ± 0.38	Flavored Milk	1.5 ± 0.32
9	Sugars	4.5 ± 1.07	Coffee, Tea	3.2 ± 1.05	Mixed Dishes-Sandwiches	3.1 ± 0.92	Flavored Milk	2.4 ± 0.65	Flavored Milk	3.9 ± 0.72	Quick Breads, Bread Products	2.7 ± 0.71	Breads, Rolls, Tortillas	2.2 ± 0.19	Breads, Rolls, Tortillas	1.8 ± 0.23	Quick Breads, Bread Products	1.9 ± 0.49	Breads, Rolls, Tortillas	1.4 ± 0.19
10	Coffee, Tea	4.1 ± 1.21	Mixed Dishes-Pizza	2.7 ± 0.87	Mixed Dishes-Pizza	2.8 ± 0.82	Sugars	2.2 ± 0.52	Quick Breads and Bread Products	3.5 ± 0.77	Breads, Rolls, Tortillas	2.4 ± 0.24	Quick Breads, Bread Products	2.1 ± 0.38	Condiments, Sauces	1.5 ± 0.19	Breads, Rolls, Tortillas	1.8 ± 0.18	Quick Breads and Bread Products	1.2 ± 0.44

[1]Mean ± standard error

Table S3. Top Ten Food Sources of Added Sugars for Adults ≥ 19 y by Ten Deciles of Intake[1]

Rank	Decile 1		Decile 2		Decile 3		Decile 4		Decile 5		Decile 6		Decile 7		Decile 8		Decile 9		Decile 10	
	Food Sub Group	% Total Added Sugars	Food Sub Group	% Total Added Sugars	Food Sub Group	% Total Added Sugars	Food Sub Group	% Total Added Sugars	Food Sub Group	% Total Added Sugars	Food Sub Group	% Total Added Sugars	Food Sub Group	% Total Added Sugars	Food Sub Group	% Total Added Sugars	Food Sub Group	% Total Added Sugars	Food Sub Group	% Total Added Sugars
1	Breads, Rolls, Tortillas	20.04 ± 0.91	Sweet Bakery Products	13.3 ± 1.29	Sweet Bakery Products	13.9 ± 1.20	Sweet Bakery Products	17.00 ± 1.11	Sweetened Beverages	23.05 ± 1.38	Sweetened Beverages	24.25 ± 1.83	Sweetened Beverages	30.84 ± 1.51	Sweetened Beverages	38.7 ± 1.83	Sweetened Beverages	45.3 ± 1.27	Sweetened Beverages	51.4 ± 1.50
2	Sweet Bakery Products	10.37 ± 0.92	Breads, Rolls, Tortillas	12.6 ± 1.18	Sweetened Beverages	11.3 ± 0.97	Sweetened Beverages	16.8 ± 1.61	Sweet Bakery Products	13.9 ± 1.14	Sweet Bakery Products	15.1 ± 0.88	Sweet Bakery Products	14.5 ± 0.91	Sweet Bakery Products	12.6 ± 0.84	Sweet Bakery Products	12.7 ± 0.94	Coffee and Tea	10.6 ± 0.96
3	Fats and Oils	9.7 ± 0.94	Sweetened Beverages	8.7 ± 1.12	Sugars	8.9 ± 0.57	Sugars	8.1 ± 0.67	Sugars	9.5 ± 0.60	Other Desserts	8.2 ± 1.29	Sugars	7.9 ± 0.97	Coffee, Tea	9.0 ± 1.36	Sugars	7.1 ± 0.75	Sweet Bakery Products	9.5 ± 0.58
4	Ready-to-Eat Cereals	7.2 ± 0.95	Sugars	8.6 ± 1.01	Breads, Rolls, Tortillas	8.4 ± 0.62	Candy	7.8 ± 0.99	Other Desserts	6.8 ± 0.96	Sugars	7.9 ± 0.77	Other Desserts	7.7 ± 1.07	Sugars	7.9 ± 0.66	Coffee, Tea	6.7 ± 0.70	Sugars	6.4 ± 0.38
5	Sugars	5.8 ± 0.80	Ready-to-Eat Cereals	7.9 ± 0.95	Candy	7.1 ± 0.84	Other Desserts	7.0 ± 0.79	Candy	6.6 ± 0.85	Coffee, Tea	6.4 ± 1.04	Candy	6.4 ± 0.98	Candy	5.4 ± 0.78	Other Desserts	4.7 ± 0.53	Candy	4.8 ± 0.44
6	Candy	5.5 ± 1.05	Fats and Oils	6.4 ± 0.90	Ready-to-Eat Cereals	6.7 ± 0.69	Breads, Rolls, Tortillas	6.2 ± 0.34	Coffee, Tea	6.1 ± 0.74	Candy	6.1 ± 0.66	Coffee, Tea	6.3 ± 0.86	Other Desserts	4.5 ± 0.55	Candy	4.5 ± 0.30	Other Desserts	4.5 ± 0.63
7	Sweetened Beverages	4.4 ± 0.75	Other Desserts	5.7 ± 0.96	Other Desserts	6.1 ± 0.81	Ready-to-Eat Cereals	5.9 ± 0.70	Breads, Rolls, Tortillas	5.8 ± 0.46	Ready-to-Eat Cereals	5.3 ± 0.61	Ready-to-Eat Cereals	3.7 ± 0.50	Ready-to-Eat Cereals	3.1 ± 0.46	Ready-to-Eat Cereals	2.8 ± 0.39	Ready-to-Eat Cereals	2.2 ± 0.17
8	Condiments and Sauces	3.8 ± 0.61	Candy	5.6 ± 0.62	Fats and Oils	5.2 ± 0.91	Coffee, Tea	4.2 ± 0.69	Ready-to-Eat Cereals	5.3 ± 0.73	Breads, Rolls, Tortillas	3.8 ± 0.33	Breads, Rolls, Tortillas	3.2 ± 0.20	Breads, Rolls, Tortillas	2.5 ± 0.21	Breads, Rolls, Tortillas	2.3 ± 0.27	Fats and Oils	1.2 ± 0.18
9	Mixed Dishes - Sandwiches	3.2 ± 0.69	Quick Breads, Bread Products	2.8 ± 0.49	Coffee, Tea	3.5 ± 0.58	Fats and Oils	3.3 ± 0.28	Fats and Oils	3.4 ± 0.38	Fats and Oils	2.6 ± 0.35	Fats and Oils	2.4 ± 0.25	Fats and Oils	2.0 ± 0.28	Quick Breads, Bread Products	1.3 ± 0.17	Breads, Rolls, Tortillas	1.1 ± 0.06
10	Cured Meats/ Poultry	2.8 ± 0.47	Condiments and Sauces	2.8 ± 0.42	Quick Breads, Bread Products	3.3 ± 0.68	Condiments and Sauces	1.9 ± 0.29	Snack/ Meal Bars	2.0 ± 0.43	Yogurt	2.6 ± 0.55	Quick Breads, Bread Products	1.8 ± 0.26	Quick Breads, Bread Products	1.4 ± 0.36	Fats and Oils	1.3 ± 0.21	Quick Breads and Bread Products	1.1 ± 0.18

[1]Mean ± standard error
